# Neratinib is an MST1 inhibitor and restores pancreatic β-cells in diabetes

**DOI:** 10.1038/s41420-019-0232-0

**Published:** 2019-12-05

**Authors:** Amin Ardestani, Matthew S. Tremblay, Weijun Shen, Kathrin Maedler

**Affiliations:** 1 Centre for Biomolecular Interactions Bremen, University of Bremen, Bremen, Germany; 2 Calibr at Scripps Research, La Jolla, CA USA

**Keywords:** Type 2 diabetes, Type 1 diabetes

The failure of pancreatic insulin-producing β-cells is a central pathogenic hallmark of all forms of diabetes. Ninety-nine years after the discovery of insulin by Banting and Best, insulin is still the only available therapy for patients with diabetes once β-cell function is fully declined. We have not succeeded yet to establish a therapeutical intervention, which maintains and restores β-cell survival and function. Pathways and targets, which control stress response, cellular homeostasis, and the apoptosis network are consequently paralleled with tumor checkpoints and the risk to exploit them for the therapy of a chronic disease such as diabetes has so far outbalanced the promises of harnessing the power of an antiapoptotic strategy to target diabetes. At the cellular and organismal level, developmental, regenerative, antiapoptotic, as well as oncogenic pathways all share some common signaling hubs and regulatory networks. Thus, their deep understanding is absolutely required to safely target and monitor such pathways in the context of therapies.

Decorated with multiple pattern recognition receptors, the pancreatic β-cell is highly sensitive to apoptotic stimuli. The necessity to survive periods of malnutrition and starvation during human evolution required highly regulated insulin secretion in tight adaptation to glucose and nutrient status. Now, overnutrition and physical inactivity fostered by Western diet and lifestyle abnormally alter glucose metabolism and energy homeostasis for which more and/or highly functional β-cells are needed to handle such metabolic imbalance. This progressive and constant high insulin demand from the β-cell leads to stress, β-cell overwork and exhaustion, degranulation, β-cell degeneration, and finally death resulting in progressive development of the relative insulin deficiency, unable to maintain normoglycemia and consequently to type 2 diabetes (T2D). Very similar in terms of consequences for the β-cell, β-cell death is thought to initiate and exacerbate immune-mediated type 1 diabetes (T1D), indicating the importance of β-cell loss in the process of T1D onset and progression.

The identification of relevant molecular pathways and pathophysiological events that are responsible for β-cell demise in diabetes is instrumental for the better understanding of disease mechanisms and to ultimately address what is truly missing in diabetes: the β-cell. Through in-depth studies of key elements of the apoptotic machinery, we have previously found serine/threonine kinase mammalian sterile 20-like kinase 1 (MST1), a core kinase of the Hippo developmental pathway, as a critical regulator of β-cell death and dysfunction in diabetes. Activated by multiple diabetogenic stimuli in human islets in vitro, in animal models of diabetes in vivo, as well as in pancreas sections obtained from patients with T2D, MST1 directly induces β-cell death and impairs insulin secretion^1^ (Fig. 1). The importance of MST1 as a therapeutical target in diabetes has been confirmed at the level of the β-cell as well as for diabetic complications, i.e., nephropathy and cardiomyopathy^2^.

**Fig. 1:**
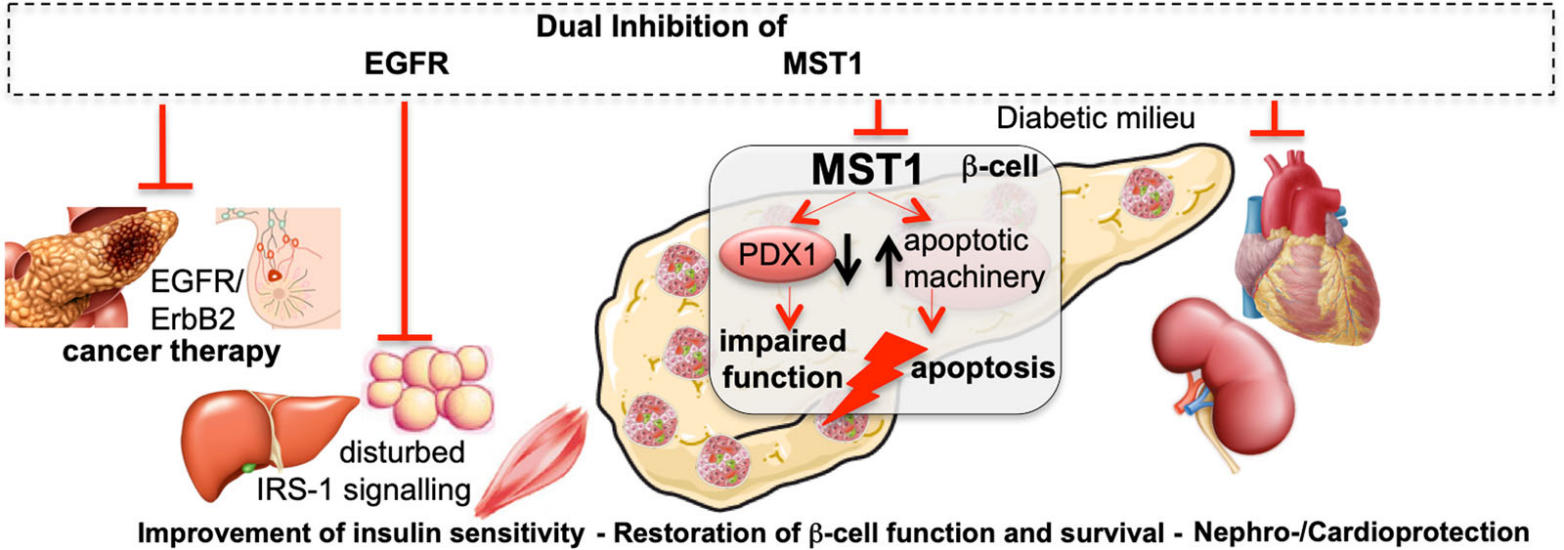
Potential effects of EGFR and MST1 signalling on metabolic disease. Targeting both EGFR/ErbB2 and MST1 has dual effects on ErbB2-sensitive cancers as well as on the metabolic syndrome: insulin sensitivity, β-cell function/survival, and diabetic complications (nephropathy and cardiomyopathy). Created using smart servier medical art under https://creativecommons.org/licenses/by/3.0/.

Since genetically targeted MST1 deficiency restores normoglycemia and β-cell function and prevents the development of diabetes^1^, we aimed in a subsequent study to find a pharmacological MST1 inhibitor with similar β-cell-protective actions. With the strategy of repurposing FDA-approved drugs for the therapy of diabetes, we performed a high-throughput MST1 inhibition screen across a highly privileged collection of 641 drug-like kinase inhibitors, together with a triaging strategy for selective, non-cytotoxic compounds, and identified neratinib, approved for breast cancer and in clinical studies for lung, colorectal, and bladder cancers targeting human epidermal growth factor receptor 2 (EGFR2, also named HER2 and ErbB2) and EGFR dual kinases, as potent MST1 inhibitor^3^. Indeed, neratinib improves β-cell survival in vitro under multiple diabetogenic conditions in β-cells and primary human and mouse islets, an effect that was specifically dependent on MST1 inhibition. Without any glucose lowering or β-cell effects in control mice, neratinib restores normoglycemia and β-cell function, survival, and mass (Fig. 1), as well as β-cell identity in the tested type 1 and 2 diabetic mouse models, namely multiple low-dose streptozotocin (MLD-STZ) and obese diabetic Lepr^db/db^ mice. MALDI imaging mass spectrometry (MALDI-IMS) shows neratinib distributed throughout the pancreas after i.p. injection. Neratinib’s effect was further confirmed in a therapeutic approach; it fully restores β-cell survival in isolated mouse islets from severely diabetic db/db mice, as well as in pro-inflammatory cytokine-treated mouse islets.

Our study focuses on the MST1-dependent effect of the ErbB2/EGFR receptor inhibitor neratinib on the β-cell. However, an important link of EGFR and diabetes has been identified before: elevated levels of ErbB2 are associated with hyperglycemia and impaired insulin sensitivity. ErbB2 reduction after weight loss indicates that it participates in the vicious cycle of the pathophysiology of diabetes. In a recent population-based cohort study with 4220 participants, high levels of ErbB2 correlated significantly with a higher risk of diabetes^4^. Therefore, such multitarget tyrosine inhibitors, initially developed for their potent anticancer effects, have been previously proposed for the treatment of diabetes, such as the potent EGFR inhibitors erlotinib and PD153035 with robust antihyperglycemic effects^5^. EGFR signaling is associated with insulin resistance and inflammation in the liver, muscle, and adipose tissue. Correspondingly, EGFR inhibition could substantially improve insulin sensitivity by reducing inflammation in insulin target tissue in obese mice, where EGFR inhibition enhances IRS-1 signaling, providing a potential therapeutic target for insulin resistance (Fig. 1). Furthermore, diabetic complications, such as cardiovascular disease and nephropathy, are also associated with EGFR signals^4^. Thus, neratinib may have positive polypharmacological effects in the setting of diabetes.

Aging, obesity, western diet, and lifestyle are the well-known causes of the severe comorbidity of cancer and diabetes. The combination of therapeutically targeting an oncogenic pathway, which is immediately connected to metabolic disease, has thus high potential. Indeed, two independent studies show profound reduction of fasting glucose levels and normalization of HbA1c in two lung cancer patients with severe T2D treated with the EGFR inhibitor erlotinib^6,7^. A hallmark observation in pancreatic cancer management is that neratinib reduces K-RAS hyperactivity, the common cancer mark, through inhibition of MST1/3/4 and Hippo signaling^8^; this makes neratinib a potent option for the therapy of the diabetes–pancreas cancer comorbidity.

Nevertheless, neratinib is cautioned with serious gastric intestinal side effects, likely due to its on-target inhibition of EGFR. Thus, part of our ongoing work is focused on the development of selective MST1 inhibitors with reduced inhibition on EGFR. In addition, MST1 inhibition by another pharmacological inhibitor (XMU-MP-1) demonstrated potential benefit in diseases beyond diabetes, for example, inflammatory bowel disease (IBD), liver injury^9^, and heart failure^10^ via enhanced tissue repair and regeneration. We will be keen on evaluating the second-generation MST1 inhibitors derived from neratinib in diabetes, heart failure, liver regeneration, and IBD.

A potential side effect in targeting MST1 for autoimmune T1D lies in the development of lymphopenia, granulocytopenia, and immunodeficiency reported in patients with biallelic MST1 deficiency^11^; however, signs of immunodeficiency, infections, or lymphopenia have not been described under neratinib therapy in a recent systematic review^12^. MST1 evokes various signaling events with opposite effects in immune and nonimmune systems by regulating immunity as well as balancing inflammation. Therefore, therapeutic MST1-depletion strategies may be promising, once they do not lead to full MST1 deficiency, which is unlikely to be achieved by pharmacological intervention.

Taken together, we show that neratinib is a previously unrecognized inhibitor of MST1 and represents a potential β-cell-protective drug with robust proof of concept in vitro in human islets and in vivo in rodent models of both T1D and T2D. Multiple effects on insulin sensitivity, diabetic complications together with the anticancer efficacy point to the high potential of targeting both the MST and EGFR/ErbB2 axes for diabetes therapy.

## References

[1] Ardestani A (2014). MST1 is a key regulator of beta cell apoptosis and dysfunction in diabetes. Nat. Med..

[2] Ardestani A, Maedler K (2018). The hippo signaling pathway in pancreatic beta-cells: functions and regulations. Endocr. Rev..

[3] Ardestani A (2019). Neratinib protects pancreatic beta cells in diabetes. Nat. Commun..

[4] Muhammad IF (2019). Circulating HER2/ErbB2 levels are associated with increased incidence of diabetes: a population-based cohort study. Diabetes Care.

[5] Fountas A, Diamantopoulos LN, Tsatsoulis A (2015). Tyrosine kinase inhibitors and diabetes: a novel treatment paradigm?. Trends Endocrinol. Metab..

[6] Costa DB, Huberman MS (2006). Improvement of type 2 diabetes in a lung cancer patient treated with Erlotinib. Diabetes Care.

[7] Brooks MB (2012). Erlotinib appears to produce prolonged remission of insulin-requiring type 2 diabetes associated with metabolic syndrome and chronic kidney disease. Br. J. Diabetes Vasc. Dis..

[8] Dent P (2019). Neratinib inhibits Hippo/YAP signaling, reduces mutant K-RAS expression, and kills pancreatic and blood cancer cells. Oncogene.

[9] Fan F (2016). Pharmacological targeting of kinases MST1 and MST2 augments tissue repair and regeneration. Sci. Transl. Med..

[10] Triastuti E (2019). Pharmacological inhibition of Hippo pathway, with the novel kinase inhibitor XMU-MP-1, protects the heart against adverse effects during pressure overload. Br. J. Pharm..

[11] Abdollahpour H (2012). The phenotype of human STK4 deficiency. Blood.

[12] Tao Z (2019). Safety and efficacy profile of neratinib: a systematic review and meta-analysis of 23 prospective clinical trials. Clin. Drug Investig..

